# Peripapillary Vascular Density in Childhood Glaucoma: A Pilot Comparative Study with Age and Sex Matched Healthy Subjects

**DOI:** 10.3390/jcm12226982

**Published:** 2023-11-08

**Authors:** Flora Xydaki, Paula Arribas-Pardo, Barbara Burgos-Blasco, Julian Garcia-Feijoo, Carmen Mendez-Hernandez

**Affiliations:** 1 Department of Inmunology, Opthalmology and ORLIIORC, Complutense University of Madrid, 28040 Madrid, Spainjgarciafeijoo@hotmail.com (J.G.-F.); 2 Ophthalmology Department, Central Defense Hospital “Gomez Ulla”, 28047 Madrid, Spain; 3 Ophthalmology Department, Hospital Clinico San Carlos, Institute of Health Research (IdISSC), 28040 Madrid, Spain

**Keywords:** childhood glaucoma, congenital glaucoma, ocular blood flow, optic nerve head, optical coherence angiography, peripapillary nerve fiber layer

## Abstract

Purpose: The aim of this study concerns the evaluation of peripapillary vessel indices in childhood glaucoma (CG) and healthy subjects. Material and Methods: In this prospective, unicenter, observational cross-sectional study, patients with CG and age and sex-matched healthy subjects were included. We compared retinal nerve fiber layer (RNFL) measurements in optical coherence tomography (OCT), peripapillary vessel density (PVD), and the flux index (FI) of the superficial vascular plexus from OCT angiography (OCT-A) between CG patients and control groups. Results: We included 39 patients (68 eyes) with CG and 50 (95 eyes) healthy subjects. The peripapillary RNFL thickness, vessel density, and flux index were significantly lower in the CG group than in the control group. The mean PVD of CG patients was 0.52 ± 0.043%, compared with 0.55 ± 0.014%, *p* < 0.0001 in healthy subjects. The mean FI was 0.32 ± 0.054 versus 0.37 ± 0.028, *p* < 0.0001, in CG patients and healthy subjects, respectively. PVD and FI in the superior, inferior, and temporal sectors were significantly lower in CG. The peripapillary RNFL thickness showed a higher area under the ROC curve (AUROC) for discriminating healthy and CG eyes and was significantly different than the PVD (0.797, 95%CI 0.726–0.869; *p* < 0.0001 vs. 0.664, 95%CI 0.574–0.752; *p* 0.00037), *p* 0.012.Conclusions: PVD and FI show lower values in CG and correlate with RNFL thickness measurement but have lower diagnostic ability than RNFL thickness measurement. Our results reveal possible differences in the pathogenesis of microvascular compromise in childhood glaucoma patients.

## 1. Introduction

Glaucoma is a neurodegenerative disease in which the death of retinal ganglion cells leads to progressive damage to the optic nerve [1]. Glaucoma is the main cause of permanent blindness due to visual field loss [2]. Previous studies in glaucoma patients have reported reduced retinal microvascularisation in peripapillary and macular areas, with vessel density loss depending on the severity of glaucoma. In order to distinguish glaucoma from normal subjects, several studies have shown that Optical Coherence Tomography Angiography (OCT-A) measurements of peripapillary and macular vessel density are comparable with Optical Coherence Tomography (OCT) measurements of the retinal nerve fiber layer thickness (RNFL) and ganglion cell thickness. A decreased density of peripapillary and macular vessels and drop out of the choroidal vasculature are also associated with the progression of the disease [3,4]. These studies have also focused on different regions of interest to distinguish the most altered areas to diagnose and detect glaucoma progression. It has been concluded that measurements at the superficial peripapillary layer are the most accurate for the assessment of glaucoma. This is because glaucomatous optic nerve axon loss, which traverses the retinal surface and coalesces in the optic nerve, makes papillary analysis the most accurate scan to collect information from all of them at the same time [5].

Childhood glaucoma is a potentially blinding condition, and it is responsible for 5% of childhood blindness worldwide [6]. Published studies show that prognosis depends on diagnosis at an early stage and adequate treatment to achieve not only intraocular pressure control but also to prevent amblyopia [7,8].

Although some types of childhood glaucoma may be easier to diagnose than adult glaucoma due to the clinical findings that children with glaucoma may present, in patients in whom the abnormality of the anterior chamber is not pronounced, symptoms are of late-onset. This is the case in patients with late-onset primary congenital glaucoma and juvenile open-angle glaucoma. In these cases, if glaucoma is suspected, a diagnosis must be confirmed, i.e., the presence of at least two of the five diagnostic criteria for childhood glaucoma must be confirmed [6]. For this reason, childhood glaucoma diagnosis can be challenging. Optic nerve head (ONH) examination is often the only feasible method to diagnose structural damage, as visual field examination is not always possible [9]. The use of OCT and other structural diagnostic tools to measure glaucomatous damage could be very helpful, but obtaining ONH images of enough quality to be assessed is not always possible, given the low visual acuity that childhood glaucoma patients may have [10,11]. Indeed, the criteria used in many studies to assess long-term outcomes in childhood glaucoma usually include intraocular pressure (IOP) monitoring, changes in the cup-to-disc ratio values, and visual acuity measurements [12].

The underlying causes of the ONH glaucomatous damage are still unclear. The mechanical and vascular theories may be related [13,14].

While there are many studies analyzing the ONH blood supply and peripapillary vascular density in adult glaucoma patients, there is not enough evidence regarding the peripapillary vascularisation in childhood glaucoma patients.

The purpose of this pilot study concerns the evaluation of the superficial peripapillary vascular density in young patients with childhood glaucoma and healthy age- and sex-matched controls.

## 2. Material and Methods

### 2.1. Study Design

This was a prospective, unicenter, observational cross-sectional study involving patients with childhood glaucoma and healthy controls, matched for age and sex, and recruited between February 2023 and June 2023 at Clinico San Carlos Hospital, Madrid, Spain.

Parents of the participants signed the informed consent form, along with patients aged 12 years and older. This study was approved by the Institutional Review Board of the Hospital Clinico San Carlos of Madrid, and the study protocol adhered to the principles of the Declaration of Helsinki.

### 2.2. Study Participants

Childhood glaucoma patients who attended glaucoma consultation for a routine exam who met the inclusion criteria were eligible for inclusion. Each participant was evaluated by experienced childhood glaucoma ophthalmologists.

Exclusion criteria for all participants were retinal pathologies, corneal, crystalline, or vitreous opacities, nystagmus, and inability to understand the purpose of the study and to consent to participation.

### 2.3. Examination Protocol

After giving informed consent on the same day, the recruited subjects underwent a complete ophthalmologic examination, including refraction and keratometry (Huvitz Auto-Ref/Keratometer HRK-7000 (Huvitz Co., Ltd., Anyang, Korea), BCVA measurement, slit-lamp biomicroscopy examination, ultrasound pachymetry (Pocket II de Quantel medical (Lumibird Medical, Lannion, France), IOP measurement using rebound tonometry iCare 200 (iCare, Tiolat Oy, Helsinki, Finland), non-mydriatic retinography (Canon CR-DGi, Canon, Tokyo, Japan), peripapillary RNFL thickness using Spectral Domain OCT (SD-OCT) Spectralis (Heidelberg Engineering, Heidelberg, Germany).

Superficial peripapillary vessel density was automatically measured using Plex Elite, applying the algorithm of the AngioPlex Elite 9000 (AngioPlex Elite 9000, Zeiss, Germany).

Visual field testing was performed only for glaucoma patients using the TOP G1 program of the Octopus perimeter (Octopus 9000 perimetry, Haag Streit AG, Bern, Switzerland).

### 2.4. OCT and OCT-A Examination

The device used for the scans was the Plex Elite 9000 (PE 9000-0180, Carl Zeiss Meditec, Dublin, CA, USA), using the AngioPlex Elite 9000 algorithm (AngioPlex Elite 9000, Zeiss, Germany). The procedure used, and the parameters measured with both SD-OCT and OCT-A are similar to those we have performed in a previous study.

The OCT-A images were obtained by one examiner (FX). Only images of good quality, as determined by a signal quality > 7/10, were included in the study.

**Figure 1:**
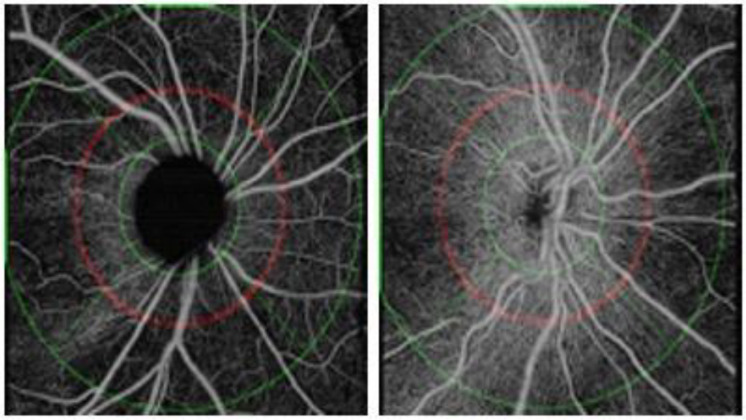
Enface optical coherence tomography angiography images (EF-OCT) of patients with infantile glaucoma (**left**) and healthy control (**right**), respectively.

Peripapillary OCT-A scans of 6 × 6 mm were performed, centered on the optic nerve head, and the SS-OCT-A device was used to obtain the enface optical coherence tomography angiography images (EF-OCT) (Figure 1).

The algorithm used to analyze the scans was the Peripapillary Nerve Fiber Layer Microvasculature Density v 0.9 from the Advanced Research and Innovation Network (ARINet). This algorithm quantifies the microcirculation within the peripapillary nerve fiber layer. More specifically, it calculates perfusion density and flow rate from the optic disc-centered OCT angiography dataset. The algorithm analyzes OCT-A images obtained from the PLEX Elite 9000, 6 × 6 mm and larger. It applies an automatic segmentation of the internal limiting membrane (ILM) and RNFL, outputs the radial peripapillary capillary vasculature (RPC) EF-OCT, and calculates capillary density metrics over an annulus centered on the optic disc. Output values are provided using both the TomTec segmentation algorithm and the multilayer segmentation algorithm. It should be noted that large retinal vessels are removed when quantifying microcirculation.

Capillary perfusion density and capillary flux density are automatically provided by the device in the area located between an inner and an outer circle, as shown in Figure 1. The two peripapillary parameters are also provided in four sectors: superior, inferior, nasal, and temporal. Perfusion density is the total area of perfused radial peripapillary capillary vasculature per unit area in a specific region (%), and the flux index is a dimensionless parameter between 0 and 1. In addition, the algorithm calculates the RNFL average thickness in microns. The average of the peripapillary RNFL thickness, as well as the average of measurements in the four quadrants, superior, inferior, nasal, and temporal, are provided. The RNFL measurements are also provided according to the Garway–Heath distribution map corresponding to the visual field sectors [15].

### 2.5. Structural and Vasculature Correlations

We compared the structure and vasculature function correlations by comparing the peripapillary RNFL thickness and peripapillary vessel density.

### 2.6. Statistical Analysis

For statistical analysis, the SPSS software package (Statistical Package for Social Sciences, v25.0; SPSS Inc., Chicago, IL, USA) was used.

Data from both eyes and each eye of all subjects included in the study were analyzed separately to avoid selection bias.

Quantitative data were tested for normal distribution using the Kolmogorov–Smirnov test.

Quantitative parameters are shown as mean and standard deviation except for parameters that did not follow a normal distribution, in which case they are expressed as median and interquartile ranges. Qualitative data are represented as their frequency distribution. Differences between the two study groups were assessed using the Student’s *t*-test for independent samples for quantitative variables and the Chi-square test in pairwise comparison for qualitative variables. Non-parametric tests were used for parameters that did not follow a normal distribution.

Pearson’s and Spearman’s correlation coefficients were used to analyze correlations between parameters.

To assess the diagnostic ability of each procedure, areas under the receiver operating characteristics curves (AUROC) were calculated. The AUROCs were compared using DeLong’s method [16].

Significance was established at *p* < 0.05.

## 3. Results

### 3.1. Analysis of Demographics

A total of 100 subjects were included in the study: 50 patients with childhood glaucoma, of whom 28 (56%) were male, and 50 age and sex-matched healthy controls. Of these 200 eyes of the 100 subjects who agreed to participate and were included in the study, 32 eyes of 11 childhood glaucoma and 5 eyes of 5 healthy controls were eliminated from the final analysis because ONH images could not be obtained, therefore ONH images were not available for OCT-A and SD-OCT analysis. The main reasons for not being able to acquire images of adequate quality were the presence of nystagmus and media opacity, the inability to maintain prolonged stable fixation due to low visual acuity, and a lack of collaboration.

Hence, data from 163 eyes, 68 eyes of 39 childhood glaucoma patients, and 95 eyes of 50 healthy control subjects were included in the final statistical analysis.

Table 1 shows the demographic data of the study participants and the results of the peripapillary vascular density analysis performed with OCT-A and the peripapillary nerve fiber layer measurements provided by Plex Elite applying the AngioPlex Elite 9000 algorithm.

Table 1A shows the ocular data for both eyes. In Table 1B and Table 1C, the results obtained from the right and the left eyes, respectively, are shown.

### 3.2. Qualitative Analysis

Most of the patients with childhood glaucoma, 30 patients, were diagnosed with primary congenital glaucoma, two with juvenile open-angle glaucoma, three with Sturge–Weber syndrome, two with juvenile idiopathic arthritis, one with Axenfeld–Rieger anomaly and one with Weill–Marchesani syndrome.

All pediatric glaucoma patients were diagnosed and treated in our hospital, and most of them had more than five years of follow-up. A total of 39 of the 68 glaucomatous eyes included in the study (57.3%) were receiving ocular hypotensive treatment, and 41 eyes (25.2%) had undergone glaucoma surgery. In addition, the group of glaucomatous patients included two eyes that had undergone cataract surgery (2.9%).

No differences were found in the distribution by sex, age, percentage of pseudophakic patients, refractive error, and pachymetry. Both groups were predominantly female. The mean age of the childhood glaucoma patient group was 13 years, while the mean age of the control group was 12 years.

### 3.3. Quantitative Analysis

Patients with glaucoma had higher IOP than the control group and significantly lower RNFL as determined by SD-OCT Spectralis. The visual field mean defect in the childhood patients’ group was 6.5 dB.

Patients with childhood glaucoma had lower values of peripapillary vascular density parameters, both for peripapillary perfusion density average and blood flux index, in both eyes and each eye independently.

The mean peripapillary vascular density of childhood glaucoma patients was 0.52 ± 0.043%, compared with 0.55 ± 0.014%, *p* < 0.0001 of healthy subjects.

The mean flux index was 0.32 ± 0.054 versus 0.37 ± 0.028, *p* < 0.0001, in childhood patients and healthy subjects, respectively. For all peripapillary quadrants, the differences in both vessel density and peripapillary flux measurements were significant, except for the nasal quadrant (Table 1A). Concerning the left eyes, although flow and vessel density were lower in all sectors, differences were not significant in the nasal, temporal, and inferior peripapillary sectors (Table 1C).

Glaucomatous eyes also had lower RNFL thickness. The mean average peripapillary RNFL thickness was 69.38 ± 14.68 vs. 83.87 ± 8.47, *p* < 0.0001 in glaucomatous subjects vs. healthy controls, while the mean average peripapillary RNFL thickness provided by the Garway–Heath sectorization measurement was also significantly lower in patients with childhood glaucoma: 78.50 ± 19.94 vs.96.70 ± 10.41, *p* < 0.0001. Significant differences were found in all analyzed RNFL thickness parameters (Table 1A).

In the subanalysis performed for each eye separately, the peripapillary RNFL was significantly lower in childhood glaucoma, although the differences in the peripapillary RNFL thickness of the temporal sector in the case of the right eyes were not significant. (Table 1B).

Table 2 shows the results of the correlations between demographic data and OCT-A vessel density parameters. Weak and negative correlations were found between age and peripapillary vessel density. Central corneal thickness showed a negative correlation with vessel parameters in the superior and inferior quadrants. No correlation was found between age and peripapillary vessel flux indices.

Table 3 shows the results of the correlation between the RNFL thickness and demographic data of the study participants.

A low positive correlation was found between the patient’s refraction sphere value and most of the peripapillary RNFL thickness measurements.

In general, correlations between peripapillary vessel density indices and patients’ ophthalmological examination data were scarce and low.

**Figure 2:**
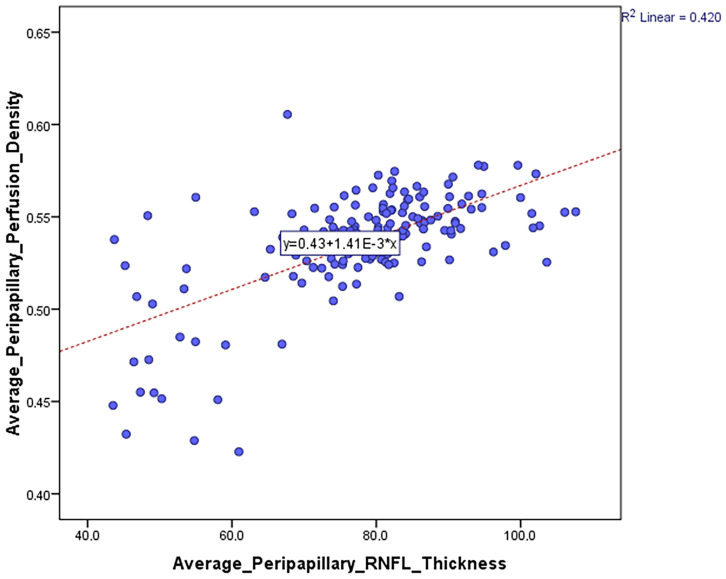
Correlation between the average peripapillary perfusion or vessel density and the average peripapillary RNFL thickness.

Figure 2 shows the correlation between the average peripapillary perfusion density and the average peripapillary RNFL thickness. A positive correlation was found (0.648, *p* < 0.0001).

**Figure 3:**
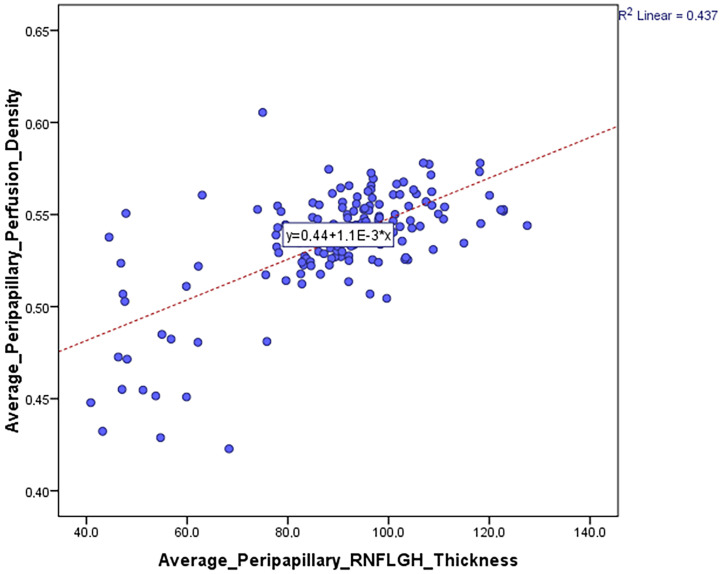
Correlation between the average peripapillary perfusion or vessel density and the mean average peripapillary RNFL thickness provided by the Garway–Heath sectorization measurement.

Figure 3 shows a positive correlation between average peripapillary perfusion density and the mean average peripapillary RNFL thickness provided by the Garway–Heath sectorization measurement (0.661, *p* < 0.0001).

Table 4 shows AUROCs for peripapillary perfusion density average, peripapillary blood flux index average, peripapillary RNFL thickness average, and peripapillary RNFL GH thickness parameters average and the cut-off point providing sensitivity at 95% and 80% specificity.

**Figure 4:**
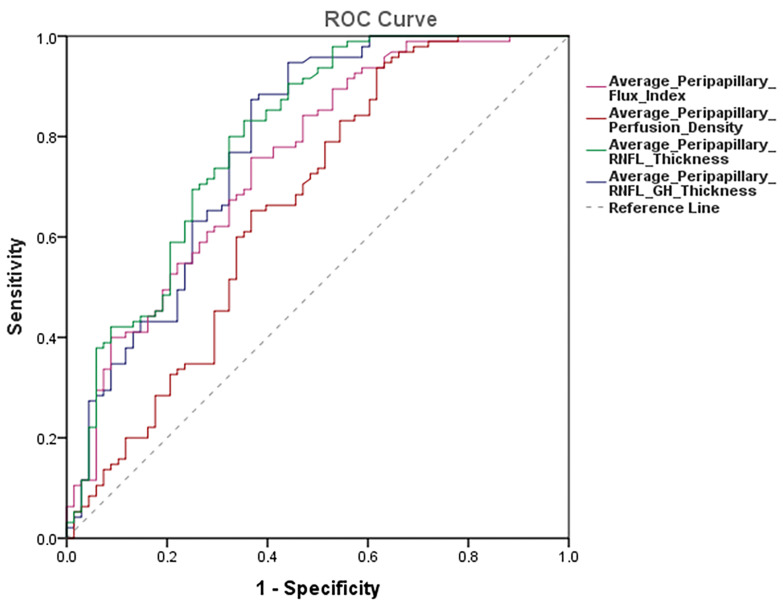
AUROCs results of the most important vascular and nerve fiber layer parameters provided by OCT-A.

Figure 4 illustrates the AUROC results of the most important vascular and nerve fiber layer parameters provided by OCT-A.

Overall, the AUROCs for discriminating between healthy and glaucomatous eyes were higher for the average peripapillary RNFL thickness with an AUROC value of 0.797 95%CI 0.726–0.869, *p* < 0.0001, followed by the average peripapillary RNFL GH thickness following the Garway–Heath distribution with an AUROC value of 0.782 95%CI 0.707–0.857, *p* < 0.0001.

The average peripapillary RNFL thickness with the Garway–Heath sectorization had the greatest sensitivity with a specificity of 95% and a sensitivity of 65% (95%CI 53 to 75%). For the specificity of 80%, there was a sensitivity of 51% (95%CI 32 to 66%) (Table 4).

Pairwise comparisons showed that AUROC of peripapillary RNFL thickness average (0.797) was significantly different than peripapillary perfusion or vessel density average (0.664), *p* 0.012, but not than peripapillary blood flux index average (0.752), *p* 0.734. Neither was there any difference between the AUROC of peripapillary RNFL average with the Garway–Heath sectorization (0.782) and peripapillary perfusion or vessel density average (0.664), *p* 0.061, or peripapillary blood flux index average (0.752), *p* 0.787. (Table 4).

## 4. Discussion

There are few studies examining peripapillary vascularization in young patients with glaucoma, hence the importance of this study. As reported in other studies, peripapillary OCT-A parameters are reduced in glaucoma patients, allowing differentiation between normal and glaucomatous eyes [3,17,18,19,20].

It has been shown that young patients with hypertensive glaucoma have reduced peripapillary perfusion density compared with normal subjects, whereas young patients with normal tension glaucoma show no difference in peripapillary vessel density compared with normal subjects. These findings suggest that initial untreated IOP values have a different impact on peripapillary vascularization [21].

In addition, a strong positive correlation has been found between the RNFL structural damage and peripapillary and ONH vascular density in juvenile open-angle glaucoma patients [22].

Our results, align with those of other studies, suggest that OCT-A can serve as a helpful tool in the diagnosis of young patients with glaucoma.

OCT-A parameters, both peripapillary vessel density and vascular flux in the superficial plexus of patients with childhood glaucoma, were lower, except for the peripapillary perfusion density in the nasal quadrant, which showed similar values in both groups. The RNFL thickness in the nasal sector is physiologically thinner than other sectors [23]. The fact that the superficial peripapillary vessel density in the nasal quadrant had been the only one without changes between the two diagnostic groups makes sense. Considering that the RNFL thickness in the superotemporal and inferotemporal sectors are most commonly damaged in the earliest stages of the disease and, therefore, show greater differences with healthy subjects, the nasal sector is less discriminatory between glaucomatous patients and healthy subjects. In fact, the thinning of the glaucomatous neuroretinal ring usually begins in the inferotemporal papillary sector and then gradually advances to the superotemporal, horizontal temporal, inferonasal, and, finally, superonasal sectors [23,24,25].

The peripapillary perfusion density average was reduced by 2% in childhood glaucoma patients, 0.52 ± 0.039, compared with healthy eyes 0.54 ± 0.015, *p* < 0.0001, and peripapillary blood flux index was reduced by 5%, 0.32 ± 0.053 versus 0.37 ± 0.031, *p* < 0.0001.

On the other hand, all RNFL thickness values, provided by both the quadrant-based instrument and the multilayer software with the sector division according to the distribution described by Garway–Heath, were significantly lower in patients with childhood glaucoma. In addition, the RNFL thickness was positively correlated with superficial peripapillary vessel density. The correlation between peripapillary perfusion density and mean peripapillary RNFL thickness provided by the Garway–Heath sectorization was r = 0.661, *p* < 0.0001.

Age was correlated with peripapillary vascular density but not with RNFL thickness. Thus, older patients had lower peripapillary vascular density. The mean age in both study groups was not statistically different, 13.28 ± 6.41 in childhood glaucoma patients vs. 12.01 ± 3.71 years old in healthy subjects, *p* = 0.169. Previous studies have shown a greater effect of age on macular than on peripapillary vessel density [18]. Our results are in agreement with those of Abay et al., who found a decrease in radial peripapillary capillary plexus vessel density with age [26].

A weak positive correlation was also found between RNFL thickness and spherical refractive error. In our study, childhood glaucoma patients were more myopic than healthy subjects. Spherical refractive errors were −2.59 ± 5.47 and −1.56 ± 2.62 in our childhood glaucoma patients and healthy controls, respectively, *p* = 0.272. This difference was expected, considering that children with childhood glaucoma have greater axial length. Patients with greater axial length are more likely to have a poorer visual prognosis. Childhood glaucoma patients with bad IOP control tend to have a greater increase in axial length and progressive myopia [27,28]. The correlation between peripapillary vessel density indices in the superficial plexus or the RNFL thickness and the visual field mean defect or loss variance was neither found nor with the visual acuity of the patients. In our study, childhood glaucoma patients had a Mean Defect of 6.5 dB and visual acuity of 0.78 ± 0.26 compared with the visual acuity of healthy subjects, which was 0.99 ± 0.12, *p* < 0.0001. Initially, we tried to include these study data from 100 eyes of 50 childhood glaucoma patients and 100 eyes of 50 normal subjects. Of these, 32 eyes from the childhood glaucoma patient group and 5 from the healthy control group were excluded due to poor OCT-A image quality, mainly caused by poor fixation, poor vision, nystagmus, and media opacity.

Therefore, patients with worse glaucoma evolution, advanced glaucoma, worse visual field results, and poor visual acuity had to be excluded. Indeed, it was to be expected, as in many cases of patients with childhood glaucoma, retinography is the only possible structural diagnostic test [6].

To our knowledge, this would be the first study that has measured the superficial peripapillary vessel density using the Angioplex^TM^ procedure, an SS-OCT-A device that obtains enface OCT images to be analyzed through the Advanced Research and Innovation Network ARINet platform. It would also be the first study in which both eyes of so many pediatric glaucoma patients have been examined, including a total of 68 eyes of childhood glaucoma patients and 95 age- and sex-matched healthy controls.

The measurements of vessel density and RNFL thickness were quantified blindly and independently of the examiner since the OCT-A images were uploaded to the ARINet platform, and the values were automatically provided by the device.

Although significant differences were found in the peripapillary vessel measurements of both eyes and each eye separately, the greatest diagnostic ability corresponded to the RNFL thickness parameters. Our results are consistent with other studies focused on OCT-A screening of glaucoma patients, even if performed using different devices [19,29].

Reduced macular and peripapillary vascularisation and blood flow have been demonstrated in patients with glaucoma, suggesting a relationship between retinal microvascularisation and the pathogenesis of the disease. Changes in the vascular superficial layer of the peripapillary region are the most relevant for the detection of glaucomatous vascular damage [30]. Our results suggest that RNFL thickness is a relevant disease marker. OCT-A can be a complementary diagnostic tool to other structural diagnostic instruments in clinical practice and could be useful in those situations in which OCT presents limitations. One of them is the floor effect, which is caused by the thickness of remaining non-axonal components in advanced glaucoma.

Although the abnormal peripapillary vessel density parameters may be useful in detecting childhood glaucoma, the mechanical damage of the optic nerve head and RNFL could be considered the main pathogenesis mechanism. Vascular dysregulation is rare in children, and ischemia of the ONH could occur in a chronic and stable manner.

OCT-A findings in childhood glaucoma could provide information about the aetiopathogenesis of the disease in children, although we are still far from considering these findings as biomarkers of the disease, nor are the identification of defects in the RNFL identified with OCT.

However, in clinical routine, it is recommended, whenever possible, to follow up the RNFL thickness with OCT in these patients. It is possible that in the future, OCT-A may be considered an additional device in the diagnosis and follow-up of this disease, as it also could be in adult glaucoma. However, at present, it is difficult to determine whether changes in the peripapillary radial vessel density in glaucoma patients, and specifically in childhood glaucoma, are a consequence of glaucomatous structural damage affecting the papilla and the peripapillary RNFL. Prospective studies will help to clarify this issue and the role of OCT-A in the diagnosis of glaucoma, particularly in childhood glaucoma.

Our study has limitations; only childhood glaucoma subjects were evaluated with a visual field. Another limitation is the sample size, as this was a pilot study on a rare disease. Despite the small sample size, enough statistical power, above 0.80, was achieved in the comparisons of peripapillary vessel parameters and RNFL thickness measurements.

Axial length could influence OCT imaging. This effect has been observed particularly on macular vascular density [31]. Our study focused on identifying differences in peripapillary vessel density in the superficial plexus between patients with childhood glaucoma and healthy controls, and although axial length was not measured, refractometry was. Patients with childhood glaucoma appeared to be more myopic, but there was no significant difference in refractive error between groups.

It should also be taken into account that enface OCT-A allows the identification of arteries and veins, while for small branches, identification can be less accurate. In addition, segmentation of the angiography slabs differs among the several OCT-A’s that are currently available; therefore, our results are difficult to compare with those of other studies [5].

## 5. Conclusions

In summary, peripapillary vessel density parameters measured using enface optical coherence tomography angiography images show significantly lower values compared with healthy subjects. Peripapillary vessel density correlates with structural damage in childhood glaucoma but has a lower diagnostic ability than RNFL thickness measurement. The greater diagnostic performance of RNFL thickness measurement suggests that OCT-A can be a complementary structural diagnostic device. Our results demonstrate the difference in clinical utility between OCT and OCT-A and illustrate possible differences in the pathogenesis of microvascular compromise in childhood glaucoma patients.

## Figures and Tables

**Table 1:** A. Demographic data and SD-OCT and OCT-A parameters of the subjects included in this study after analyzing the results of both eyes. B. Demographic data and SD-OCT and OCT-A parameters of the subjects included in this study after analyzing the results of the right eye. C. Demographic data and SD-OCT and OCT-A parameters of the subjects included in this study after analyzing the results of the left eye.

A			
	Normal (*n* = 95)	Glaucoma (*n* = 68)	*p* Value
Gender (M/F)	35/60	26/42	0.492 ^&^
Age (year)	12.01 ± 3.71	13.47 ± 6.42	0.069 ^#^
Patients under glaucoma medications	-	39 (57.3%)	-
Glaucoma surgery	-	41 (60.3%)	-
Pseudophakia	0 (0%)	2 (2.9%)	0.173 ^&^
Visual acuity	0.99 ± 0.12	0.78 ± 0.26	**<0.0001** ^#^
Sphere	−1.56 ± 2.62	−2.59 ± 5.47	0.153 ^#^
Astigmastism	−1.00 [−1.75;−0.50]	−1.00 [−1.75;−0.50]	0.536 *
Mean Defect (dB)	-	6.50 [1.72;10.82]	-
Loss Variance (dB^2^)	-	4.24 [2.60;5.55]	-
Intraocular Pressure (mmHg)	15.96 ± 3.36	18.60 ± 4.98	**0.015** ^#^
Pachymetry (μ)	539.68 ± 38.66	553.31 ± 48.26	0.199 ^#^
Cup-to-disk ratio	0.37 ± 0.15	0.47 ± 0.26	**0.002** ^#^
RNFL (μ)	101.38 ± 10.40	82.12 ± 24.43	**<0.0001** ^#^
Peripapillary OCT-A Vessel Density			
Average Vessel Density (%)	0.54 ± 0.015	0.52 ± 0.039	**<0.0001** ^#^
Average Blood Flux Index	0.37 ± 0.031	0.32 ± 0.053	**<0.0001** ^#^
Superior Quadrant Vessel Density (%)	0.54 ± 0.022	0.51 ± 0.057	**<0.0001** ^#^
Superior Quandrant Flux Index	0.37 ± 0.030	0.32 ± 0.052	**<0.0001** ^#^
Inferior Quadrant VesselDensity (%)	0.55 ± 0.061	0.51 ± 0.079	**0.001** ^#^
Inferior Quadrant Flux Index	0.36 ± 0.049	0.32 ± 0.066	**<0.0001** ^#^
Temporal Quadrant Vessel Density (%)	0.56 ± 0.025	0.55 ± 0.044	**0.011** ^#^
Temporal Quadrant Flux Index	0.38 ± 0.033	0.33 ± 0.058	**<0.0001** ^#^
Nasal Quadrant Vessel Density (%)	0.52 ± 0.029	0.52 ± 0.039	0.245 ^#^
Nasal Quadrant Flux Index	0.37 ± 0.034	0.32 ± 0.052	
Peripapillary RNFL Thickness			
Average RNFL Thickness (μ)	83.87 ± 8.47	69.38 ± 14.68	**<0.0001** ^#^
Average GH RNFL Thickness(μ)	96.70 ± 10.41	78.50 ± 19.94	**<0.0001** ^#^
Superior Quadrant RNFL Thickness (μ)	105.99 ± 13.09	81.65 ± 23.54	**<0.0001** ^#^
Inferior Quadrant RNFL Thickness (μ)	105.62 ± 19.32	80.28 ± 25.70	**<0.0001** ^#^
Temporal Quadrant RNFL Thickness (μ)	63.99 ± 11.95	59.45 ± 12.79	**0.021** ^#^
Nasal Quadrant RNFL Thickness (μ)	61.93 ± 9.20	56.21 ± 9.34	**<0.0001** ^#^
GH Temporal Sector RNFL Thickness (μ)	71.64 ± 15.46	65.44 ± 16.53	**0.015** ^#^
GH Superotemporal Sector RNFL Thickness(μ)	133.69 ± 24.03	98.40 ± 39.21	**<0.0001** ^#^
GH Inferotemporal Sector RNFL Thickness (μ)	145.58 ± 25.03	111.40 ± 44.62	**<0.0001** ^#^
GH Nasal Sector RNFL Thickness (μ)	76.31 ± 15.58	66.21 ± 14.10	**<0.0001** ^#^
GH Superonasal Sector RNFL Thickness (μ)	121.24 ± 26.43	90.71 ± 30.93	**<0.0001** ^#^
GH Inferonasal Sector RNFL Thickness (μ)	115.29 ± 29.74	84.53 ± 34.83	**<0.0001** ^#^
**B**			
	**Normal** **(*n* = 47)**	**Glaucoma** **(*n* = 32)**	***p* value**
Gender (M/F)	16/31	13/19	0.359 ^&^
Age (year)	12.09 ± 3.72	13.69 ± 6.52	0.169 ^#^
Patients under glaucoma medications	-	18 (56.2%)	-
Glaucoma surgery	-	19 (59%)	-
Pseudophakia	0 (0%)	2 (6.2%)	0.649 ^&^
Visual acuity	0.99 ± 0.12	0.77 ± 0.29	**<0.0001** ^#^
Sphere	−1.49 ± 2.51	−2.68 ± 6.08	0.272 ^#^
Astigmastism	−1.00 [−2.06;−0.50]	−1.00 [−2.06;−0.50]	0.875 *
Mean Defect (dB)	-	5.55 [0.45;9.35]	-
Loss Variance (dB^2^)	-	4.25 [2.60;5.55]	-
Intraocular pressure (mm Hg)	15.67 ± 3.31	18.63 ± 5.31	0.081 ^#^
Pachymetry (μ)	542.00 ± 40.86	559.79 ± 51.32	0.289 ^#^
Cup-to-disk ratio	0.39 ± 0.16	0.51 ± 0.28	**0.020** ^#^
RNFL (μ)	102.49 ± 10.91	80.56 ± 25.20	**<0.0001** ^#^
Peripapillary OCT-A Vessel Density			
Average Vessel Density (%)	0.55 ± 0.014	0.52 ± 0.043	**<0.0001** ^#^
Average Blood Flux Index	0.37 ± 0.028	0.32 ± 0.054	**<0.0001** ^#^
Superior Quadrant Vessel Density (%)	0.55 ± 0.023	0.51 ± 0.057	**<0.0001** ^#^
Superior Quadrant Flux Index	0.37 ± 0.027	0.32 ± 0.051	**<0.0001** ^#^
Inferior Quadrant Vessel Density (%)	0.55 ± 0.022	0.51 ± 0.054	**<0.0001** ^#^
Inferior Quadrant Flux Index	0.37 ± 0.030	0.32 ± 0.055	**<0.0001** ^#^
Temporal Quadrant Vessel Density (%)	0.57 ± 0.022	0.55 ± 0.047	**0.026** ^#^
Temporal Quadrant Flux Index	0.38 ± 0.028	0.33 ± 0.057	**<0.0001** ^#^
Nasal Quadrant Vessel Density (%)	0.53 ± 0.026	0.52 ± 0.042	0.277 ^#^
Nasal Quadrant Flux Index	0.37 ± 0.032	0.32 ± 0.053	**<0.0001** ^#^
Peripapillary RNFL Thickness			
Average RNFL Thickness (μ)	84.87 ± 9.04	69.97 ± 14.68	**<0.0001** ^#^
Average GH RNFL Thickness (μ)	97.67 ± 10.90	79.59 ± 20.57	**<0.0001** ^#^
Superior Quadrant RNFL Thickness (μ)	105.70 ± 13.54	80.73 ± 21.72	**<0.0001** ^#^
Inferior Quadrant RNFL Thickness (μ)	107.07 ± 16.82	79.02 ± 21.93	**<0.0001** ^#^
Temporal Quadrant RNFL Thickness (μ)	65.64 ± 12.51	61.80 ± 14.34	0.211 ^#^
Nasal Quadrant RNFL Thickness (μ)	63.58 ± 9.51	58.50 ± 9.75	**0.024** ^#^
GH Temporal Sector RNFL Thickness (μ)	72.50 ± 15.47	68.27 ± 19.31	0.284 ^#^
GH Superotemporal Sector RNFL Thickness (μ)	140.99 ± 23.16	102.33 ± 40.33	**<0.0001** ^#^
GH Inferotemporal Sector RNFL Thickness (μ)	144.71 ± 24.31	110.14 ± 44.80	**<0.0001** ^#^
GH Nasal Sector RNFL Thickness (μ)	78.08 ± 16.33	68.44 ± 14.46	**0.009** ^#^
GH Superonasal Sector RNFL Thickness (μ)	115.96 ± 28.13	87.05 ± 27.47	**<0.0001** ^#^
GH Inferonasal Sector RNFL Thickness (μ)	117.49 ± 30.77	81.17 ± 28.41	**<0.0001** ^#^
**C**			
	**Normal** **(*n* = 48)**	**Glaucoma** **(*n* = 36)**	***p* value**
Gender (M/F)	19/29	13/23	0.462 ^&^
Age (year)	11.94 ± 3.74	13.28 ± 6.41	0.233 ^#^
Patients under glaucoma medications	-	21 (58.3%)	-
Glaucoma surgery	-	22/36	-
Pseudophakia	-	-	-
Visual acuity	0.99 ± 0.12	0.77 ± 0.29	**<0.0001** ^#^
Sphere	−1.65 ± 2.75	−2.51 ± 5.07	0.369 ^#^
Astigmastism	−1.00 [−1.75;−0.50]	−1.00 [−1.75;−0.50]	0.500*
Mean Defect (dB)	-	7.15 [2.63;13.23]	-
Loss Variance (dB^2^)	-	4.80 [3.23;6.10]	-
Intraocular pressure (mm Hg)	16.21 ± 3.51	18.57 ± 4.75	0.100 ^#^
Pachymetry (μ)	537.67 ± 37.97	548.29 ± 45.98	0.443 ^#^
Cup-to-disk ratio	0.35 ± 0.15	0.44 ± 0.24	**0.036** ^#^
RNFL (μ)	100.28 ± 9.87	83.59 ± 23.96	**<0.0001** ^#^
Peripapillary OCT-A Vessel Density			
Average Vessel Density (%)	0.54 ± 0.016	0.53 ± 0.035	**0.006** ^#^
Average Blood Flux Index	0.37 ± 0.034	0.33 ± 0.054	**<0.0001** ^#^
Superior Quadrant Vessel Density (%)	0.54 ± 0.020	0.52 ± 0.058	**0.013** ^#^
Superior Quadrant Flux Index	0.37 ± 0.033	0.33 ± 0.053	**<0.0001** ^#^
Inferior Quadrant Vessel Density (%)	0.54 ± 0.083	0.51 ± 0.097	0.143 ^#^
Inferior Quadrant Flux Index	0.36 ± 0.062	0.32 ± 0.074	**0.009** ^#^
Temporal QuadrantVessel Density (%)	0.55 ± 0.025	0.54 ± 0.041	0.187 ^#^
Temporal Quadrant Flux Index	0.37 ± 0.037	0.33 ± 0.059	**<0.0001** ^#^
Nasal Quadrant Vessel Density (%)	0.52 ± 0.033	0.51 ± 0.037	0.578 ^#^
Nasal Quadrant Flux Index	0.36 ± 0.036	0.32 ± 0.051	**<0.0001** ^#^
**Peripapillary RNFL Thickness**			
Average RNFL Thickness(μ)	82.90 ± 7.83	68.85 ± 14.87	**<0.0001** ^#^
Average GH RNFLThickness (μ)	95.75 ± 9.93	77.53 ± 19.60	**<0.0001** ^#^
Superior Quadrant RNFL Thickness (μ)	106.29 ± 12.79	82.47 ± 25.32	**<0.0001** ^#^
Inferior Quadrant RNFL Thickness (μ)	104.20 ± 21.58	81.40 ± 28.91	**<0.0001** ^#^
Temporal Quadrant RNFL Thickness (μ)	62.37 ± 11.26	57.36 ± 11.01	**0.045** ^#^
Nasal Quadrant RNFL Thickness (μ)	60.31 ± 8.70	54.17 ± 8.57	**0.002** ^#^
GH Temporal Sector RNFL Thickness (μ)	70.81 ± 15.56	62.93 ± 13.37	**0.017** ^#^
GH Superotemporal Sector RNFL Thickness (μ)	126.54 ± 22.89	94.90 ± 38.41	**<0.0001** ^#^
GH Inferotemporal Sector RNFL Thickness (μ)	146.44 ± 25.96	112.53 ± 25.07	**<0.0001** ^#^
GH Nasal Sector RNFL Thickness (μ)	74.57 ± 14.77	64.24 ± 13.66	**0.002** ^#^
GH Superonasal Sector RNFL Thickness (μ)	126.41 ± 23.83	93.96 ± 33.77	**<0.0001** ^#^
GH Inferonasal Sector RNFL Thickness (μ)	113.13 ± 28.87	87.52 ± 39.84	**0.001** ^#^

^&^ Chi-square test, ^#^ t-Student test, * Median and [P25;P75]. RNFL: Average Retinal Nerve Fiber Layer. GH: Garway–Heath sectorization. All above measurements are represented by mean ± SD except Mean Defect, Loss Variance, and astigmatism, which are expressed by median and [P25;P75]. Significant *p* value are in bold.

**Table 2:** Correlation between the most relevant OCT-A vessel density and demographic data.

	Average Vessel Density (%)	Superior Quadrant Vessel Density (%)	Inferior Quadrant Vessel Density (%)	Nasal Quadrant Vessel Density (%)	Temporal Quadrant Vessel Density (%)	Superior Quadrant Flux Index	Inferior Quadrant Flux Index
Age (year)	−0.221 **(0.005) ^§^**	−0.169 **(0.031) ^§^**	−0.179 **(0.023) ^§^**	−0.207 **(0.008) ^§^**	−0.158 **(0.044) ^§^**		
Cilinder					−0.175 **(0.045)** *		
Pachymetry (μ)		−0.227 **(0.039) ^§^**	−0.295 **(0.007) ^§^**			−0.235 **(0.032) ^§^**	−0.288 **(0.009) ^§^**

^§^ Pearson correlation coefficient, * Spearman correlation coefficient. Significant *p* value in bold.

**Table 3:** Correlation between the RNFL thickness parameters and demographic data.

	Average RNFL Thickness (μ)	Superior Quadrant RNFL Thickness (μ)	Inferior Quadrant RNFL Thickness (μ)	Temporal Quadrant RNFL Thickness (μ)	Nasal Quadrant RNFL Thickness(μ)	Average GH RNFL Thickness (μ)	GH Temporal Sector RNFL Thickness(μ)	GH Inferotemporal Sector RNFL Thickness (μ)	GH Nasal Sector RNFL Thickness (μ)	GH Superonasal Sector RNFL Thickness (μ)	GH Inferonasal Sector RNFL Thickness (μ)
Glaucoma meds				−0.243 **(0.031)** *			−0.244 **(0.030)** *				
Visual acuity							0.175 **(0.031) ^§^**				
Sphere	0.204 **(0.020) ^§^**	0.214 **(0.014) ^§^**	0.260 **(0.003) ^§^**	−0.181 **(0.040) ^§^**	0.244 **(0.005) ^§^**	0.203 **(0.021) ^§^**		0.229 **(0.009) ^§^**	0.246 **(0.005) ^§^**	0.218 **(0.013) ^§^**	0.217 **(0.013) ^§^**
Cilinder										0.182 **(0.036)** *	−0.305 **(0.005)** *
Pachymetry (μ)		−0.279 **(0.011) ^§^**	−0.273 **(0.013) ^§^**								

^§^ Pearson correlation coefficient, * Spearman correlation coefficient. Significant *p* value in bold.

**Table 4:** AUROC values of the main vessel density parameters were determined with OCT-A PLEX Elite 9000.

					Sb at 95% of Sp					Sb at 80% of Sp			
	AUROC	95%CI	*p* Value	Cut Off	Sb	95%CI	LR+	LR−	Cut Off	Sb	95%CI	LR+	LR−
Vessel Density (%)	0.664	0.575; 0.752	**0.00037**	0.52	0.35	0.22; 0.47	0.83	0.67	0.53	0.47	0.34; 0.60	0.63	0.69
Blood Flux Index	0.752	0.675; 0.828	**<0.0001**	0.31	0.37	0.24; 0.51	0.84	0.68	0.35	0.56	0.41; 0.71	0.67	0.72
RNFL Thickness (μ)	0.797	0.725; 0.869	**<0.0001**	72.19	0.49	0.35; 0.62	0.87	0.72	77.00	0.66	0.51; 0.78	0.70	0.77
GH RNFL Thickness (μ)	0.782	0.707; 0.857	**<0.0001**	83.16	0.51	0.32; 0.66	0.88	0.73	88.34	0.65	0.53; 0.75	0.70	0.76

AUROC: Area under the ROC curve. Sb: Sensitivity. LR: likelihood ratio. 95%CI: 95% Confidence interval. RNFL: retinal nerve fiber layer. GH: Garway–Heath sectorization. Sb: Sensitivity. Sp: Specificity. Significant *p* value in bold.

## Data Availability

Available upon request.
